# A Case of U-shaped Sacral Fracture After Longstanding Spinopelvic Fixation Treated With Percutaneous Sacroiliac Joint Fusion and Iliosacral Osteosynthesis

**DOI:** 10.7759/cureus.47152

**Published:** 2023-10-16

**Authors:** Vinayak Ganeshan, Daniel Denis

**Affiliations:** 1 Neurosurgery, Ochsner Health, New Orleans, USA

**Keywords:** iliosacral osteosynthesis, minimally invasive surgery, sacroplasty, image-guided navigation, thoracolumbar fusion, sacral nonunion

## Abstract

Sacral fractures are pelvic ring injuries that usually occur following a fall from height and may present with neurological injury. They are divided into several subtypes based on the pattern and location of injury. Certain subtypes require operative management due to the risk of neural compromise and inadequate axial load transfer, limiting mobility. Spinopelvic fixation has been reported as an efficient surgical treatment to restore the stability of U-shaped sacral fractures and to accelerate healing by relieving sacral stress. It is unclear if low-velocity sacral fractures occurring after longstanding lumbosacral fusion with pelvic fixation require additional surgical intervention. An elderly female with osteoporosis and prior T4-pelvis instrumented fusion sustained a fragility sacral fracture and was treated conservatively. At follow-up, she developed a symptomatic U-shaped sacral fracture. The increased fracture displacement and nonunion were chiefly attributed to sacroiliac joint hypermobility. A percutaneous osteosynthesis at the S1 and S2 levels was performed with a novel type of implant to achieve concomitant sacroiliac joint stabilization and fusion. Implants were placed with the help of intraoperative three-dimensional imaging and image-guided navigation to avoid the previously installed pelvic hardware. In summary, U-shaped fractures can develop nonunion despite pre-existing spinopelvic fixation and can be treated adequately with percutaneous iliosacral osteosynthesis. A sacroiliac joint fixation and fusion should be considered in the same setting as sacroiliac joint instability may contribute to or exacerbate nonunion.

## Introduction

Sacral fractures are pelvic ring injuries that usually occur following a fall from height and may present with neurological injury [1]. There are multiple systems of classification of sacral fractures based on their morphology, the adjacent structures involved, and the displacement of the fracture components. Certain subtypes require operative intervention to prevent neural compromise and long-term disability [2]. Lumbopelvic fixation has been reported as an efficient surgical treatment to restore the stability of U-shaped fractures [3]. It is hypothesized that by transferring the load of the spine to the pelvis using instrumentation, the stress on the sacral fracture is reduced, thereby allowing it to heal. Iliosacral screw fixation using percutaneous osteosynthesis is another established method to stabilize a U-shaped sacral fracture by directly stabilizing the fracture fragments [4].

Traumatic sacral fractures occurring after thoracolumbar fusion with pelvic fixation are rare events for which management is scarcely discussed in the literature. One would assume that the prior lumbopelvic fixation would assist in the stabilization and healing of the fracture. However, the existence of prior risk factors such as osteoporosis and vitamin D deficiency can increase the risk of sacral nonunion [5]. The case presented here is a sacral insufficiency fracture progressing to an unstable U-shaped sacral fracture, despite prior sacropelvic fixation. It is hypothesized that bilateral sacroiliac (SI) joint laxity and extension of the fracture fragments to the SI joints contributed to sacral nonunion. This case was managed successfully with image-guided percutaneous placement of implants designed to achieve fracture stabilization, SI joint fixation, and fusion. This case suggests that SI joint instability can contribute to the progression of U-type facture, highlighting the importance of SI joint fixation and fusion in managing sacral fractures.

## Case presentation

**Figure 1:**
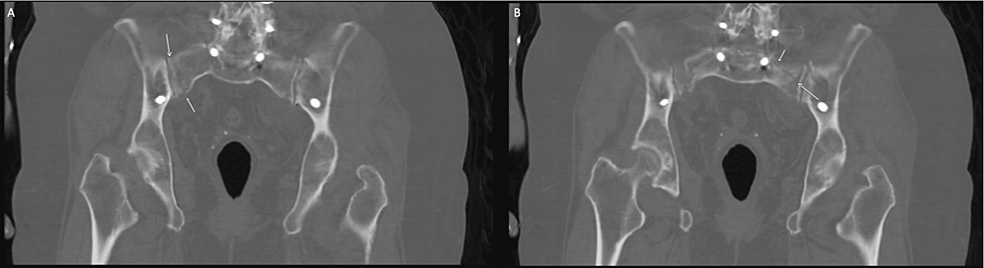
Coronal reformat of the CT scan of the pelvis performed on the day of the fall. (A) Right-sided fracture of the sacral ala and (B) left-sided fracture of the sacral ala with extension to the sacroiliac joint are demonstrated (white arrows).

The patient was a 73-year-old woman with a history of osteoporosis. She had a T4 to S1 instrumented fusion in 2007 for the treatment of idiopathic scoliosis. Following a fall from standing height, she was diagnosed with a sacral insufficiency fracture. A computed tomography (CT) scan of the pelvis revealed bilateral longitudinal sacral ala fractures, comminuted on the left side with extension to the left SI joint and S3 sacral foramina (Denis classification zone II) (Figure 1) [6]. No emergent surgical intervention was recommended by the on-call team. She was transferred to a rehabilitation facility for two-and-a-half weeks before being discharged home with home health support.

**Figure 2:**
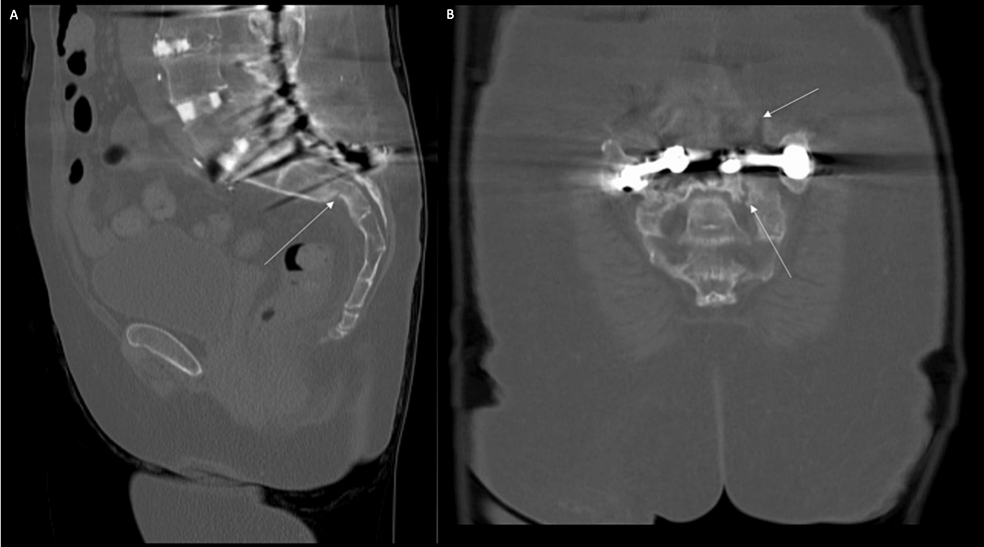
One-month follow-up sagittal view (A) demonstrating worsening sacral kyphosis (white arrow) (Roy-Camille type 1). Coronal view (B) demonstrating U-shaped morphological fracture through the S2 foramen in the setting of existing hardware (white arrows).

One month after discharge, she visited her primary care doctor complaining of worsening pain when sitting and lying down and underwent a repeat CT scan, showing nonunion and a new transverse fracture component (Denis classification zone III). Sagittal views of the CT revealed increased sacral kyphosis due to the development of a U-shape fracture (Roy-Camille classification type 1) (Figure 2) [7]. She underwent an endocrinological workup for nonunion, which was unremarkable. Due to her history of osteoporosis and the fatigue component of her fracture, a bilateral percutaneous sacroplasty using the long-axis technique [8] was performed with spinal cement (Confidence Spinal Cement System®, Johnson & Johnson, Raynham, MA) to fill the bony voids and allow for better subsequent fixation. This was followed by a bilateral percutaneous iliosacral osteosynthesis of the fracture elements using implants designed to stabilize and fuse the sacroiliac joints in the same setting (iFuse-TORQ, SI-BONE, Santa Clara, CA).

**Figure 3:**
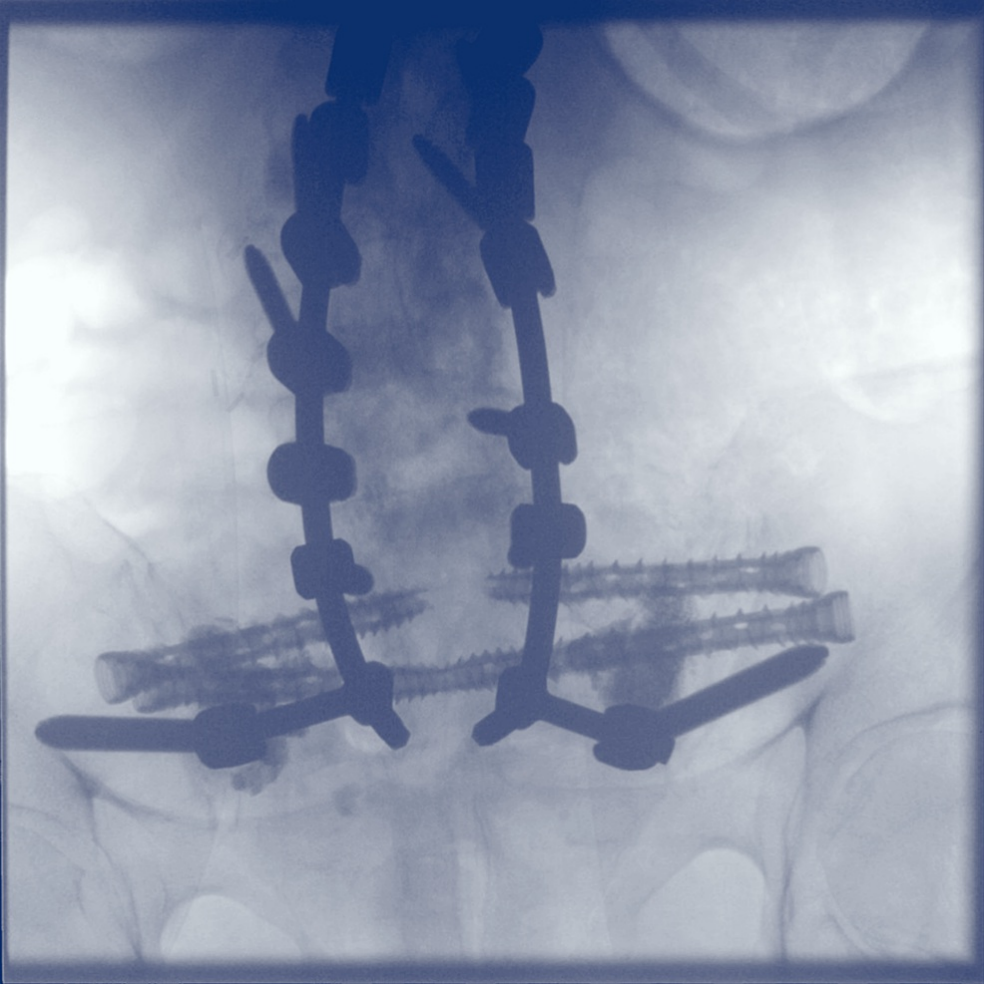
X-ray taken intraoperatively demonstrating placement of percutaneous screws spanning the sacroiliac joint and fracture lines.

Percutaneous iliosacral fixation is usually performed with fluoroscopy using inlet and outlet views [9]. Due to the presence of pelvic fixation screws impeding the iliosacral trajectory between the S1 and S2 foramen, intraoperative three-dimensional (3D) imaging (Ziehm Imaging Inc., Orlando, FL), implant planning, and image-guided navigation (Curve®, Brainlab Inc., Westchester, IL) were utilized to ensure safe and precise implant placement above the S1 foramen and between the S1 and S2 foramen bilaterally (Figure 3).

**Figure 4:**
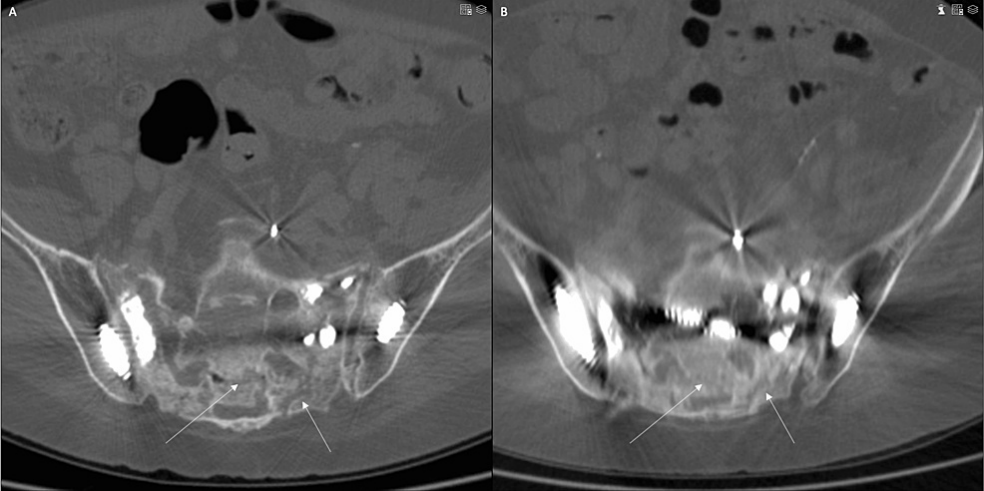
Axial computed tomography of the pelvis before (A) and 10 months after (B) iliosacral fixation and sacroiliac joint fusion. Partial consolidation of the U-shaped fracture at 10 months postoperative scan is visible (white arrows).

In the weeks following the procedure, the patient’s severe sacral pain subsided, but she complained of a new right leg pain without motor or sensory deficit. The pain was attributed to S1 nerve root irritation by the caudal right implant, which abutted (but did not violate) the right S1 foramen on postoperative imaging. She received a right-sided S1 transforaminal epidural nerve block and steroid injection, which alleviated the pain completely. Six weeks post procedure, she had complete resolution of her right leg pain and reported >75% sacral pain relief. She also reported improved mobilization postoperatively. At her three-month follow-up appointment, she reported mild residual intermittent left-sided sacral pain but continually improved functional mobility, measured by her ability to sit, stand, and walk. At her six-month follow-up, she reported minimal lower back pain. A 10-month postoperative subsequent CT scan showed both partial consolidation of the fracture and the absence of radiolucency around the hardware (Figure 4).

## Discussion

Sacral fractures typically occur in two patient populations: either in the young following high-energy trauma, or in the elderly following low-energy trauma in the setting of prior osteoporosis [2]. U-type fractures are commonly associated with some degree of neurological compromise due to the complex regional anatomy that can impinge upon or transect the nerve roots emerging from the cauda equina through the sacral foramina [10]. The gold standard diagnostic technique for sacral fractures is a CT scan; longitudinal and U-type fractures are generally best appreciated on sagittal and coronal reconstruction [11]. In the setting of suspected neural compromise, a magnetic resonance imaging (MRI) scan can further distinguish cauda equina or nerve root involvement [11]. The treatment of these fractures varies depending on the type of fracture, degree of fracture displacement, pelvic instability, and presence of neurological symptoms such as radiculopathy and/or sphincter dysfunction [12].

There are several classification systems for fractures based on their appearance and the structures involved. The Denis classification system divides longitudinal fractures based on their relationship to the sacral foramina and sacral canal [6]. The Roy-Camille classification system further subdivides zone III Denis sacral fractures into three subtypes based on the relationship of the lumbosacral spine to the distal sacral fragment in the setting of displacement [7]. A morphological system describes transverse sacral fractures based loosely on their shape into T-type, H-type, U-type, and Lambda-type fractures [13]. These classification systems help guide the course of management. The most concerning types of sacral fractures disrupt the proper force transfer from the spinal column to the sacral body and pelvis; this phenomenon is known as spinopelvic dissociation (SPD) and is an indication for operative intervention [13]. U-type sacral fractures universally require surgical management as the fracture alignment causes SPD, which can lead to increased kyphotic angulation of the caudal sacral fracture component and subsequent neural compromise [9].

Arthrodesis of the lumbosacral spine can be challenging due to the complex anatomy of the region and the sheer magnitude of biomechanical forces in play. The sacrum itself is a relatively cancellous bone that houses critical nerve roots and is adjacent to neurovascular structures responsible for the perfusion and innervation of the entire caudal region. In the setting of SPD, the goal of surgical treatment is to restore continuity and force transfer between the spine and the pelvis through fixation. For U-shaped fractures, lumbopelvic fixation has been reported as an efficient surgical treatment to restore stability [3]. Through this surgical technique, the spinal load is transferred to the pelvis using instrumentation, relieving the stress on the sacral fracture and allowing it to heal. Directly stabilizing the fracture fragments using iliosacral osteosynthesis is another method to stabilize a U-shaped sacral fracture [4]. The main advantage of this technique is that it immediately corrects SI joint instability, hypothesized as the principal reason for sacral fracture progression in the case presented. There are additional advantages to this approach; it can be performed rapidly through a percutaneous approach even in the setting of existing hardware, and it generally leads to minimal blood loss, short length of stay, and expedited recovery. For these reasons, iliosacral osteosynthesis was selected as the operation of choice for this patient. The use of intraoperative 3D imaging, planning, and navigation allowed for the precise positioning of implants around the previously installed pelvic hardware despite significant metallic artifacts. Image-guided navigation helped determine the best trajectories to achieve osteosynthesis of the sacral fracture fragments while achieving adequate SI joint stabilization. It also minimized operative time and exposure to radiation and helped decipher the intricate sacral anatomy already complicated by pre-existing hardware [14].

Sacral fractures following thoracolumbar fusion to the sacrum generally occur from a few weeks to seven months postoperatively [15]. The proposed mechanisms predisposing these patients to sacral fractures are pseudoarthrosis at L5-S1 and excessive force transmission to the sacrum. Extension of the existing fusion to the pelvis has successfully treated this type of fracture when conservative management fails [15]. However, a traumatic sacral nonunion occurring after a longstanding thoracolumbar fusion with pelvic fixation appears to be a rare event and the management of these fractures has not been previously discussed in the literature. With an increasing number of elderly patients having undergone surgery to correct spinal deformities, this presentation is likely to increase in preponderance. Early follow-up and repeat imaging should be considered in the setting of known acute sacral fracture to rule out nonunion or fracture progression. In this case, nonunion was demonstrated on follow-up CT imaging via increased anterior displacement and kyphotic angulation of the lumbosacral spine rostral to the S3 vertebral body.

Different hypotheses can explain why the fracture became unstable despite pre-existing hardware connecting the lumbosacral spine to the pelvis. The caudal fracture fragment, consisting of the bilateral sacral ala connected to the S3 vertebral body, was still connected to the pelvis by the sacroiliac joints. Long-standing lumbosacral fusion is known to increase the SI joint range of motion, which can result in pain and disability [16]. The combination of increased SI joint laxity and sacral ala fracture lines extending to the SI joints could explain why the fracture displacement worsened. It is also possible that the pre-existing spinal hardware connecting the lumbosacral spine to the pelvic bones was not perfectly rigid due to loosening at the connector sites. However, the patient did not have clinical or radiological signs of pseudoarthrosis, and no radiolucency was visible on scans of the existing hardware. The rapid improvement in the patient-reported pain and mobility following SI joint stabilization and iliosacral osteosynthesis suggests that SI joint hypermobility contributed to the fracture progression. Partial consolidation of the fracture was also radiologically demonstrated 10 months postoperatively (Figure 4).

A sacroplasty was performed prior to the iliosacral osteosynthesis. Sacroplasty is a well-described treatment for sacral insufficiency fractures and has been used for patients to improve chronic sacral fracture pain [17]. The cementation of trabecular voids adds stability and is associated with reportedly better union outcomes when used in conjunction with percutaneous screw fixation [18]. Patients who have undergone in situ fixation for nonunion have healed with high rates of reported satisfaction [19].

## Conclusions

There is a small but growing population of elderly patients with prior spinopelvic fixation who experience a sacral fracture. These patients’ prior risk factors, the morphology of their fracture, and post-fixation changes in SI joint mobility may arrest healing, leading to continued sacral nonunion. Continued surveillance is recommended to ensure that sacral fracture progression can be both diagnosed and treated promptly, as prior spinopelvic fixation is not a guarantee against the development of sacral nonunion. Extending a patient’s existing sacral thoracolumbar fusion to the pelvis is one method to successfully treat these fractures. However, in this unique case, the sacral fracture progressed despite prior pelvic fixation. This case highlights the structural causes that can lead to sacral nonunion in this population and demonstrates the advantages of iliosacral osteosynthesis to both stabilize the SI joints and reduce the stress on the fracture. Combined sacral kyphoplasty with percutaneous osteosynthesis and SI joint fusion was shown to be an effective means of stabilizing a U-type fracture with prior lumbopelvic fixation, relieving pain, and improving mobility both acutely and long term. Intraoperative 3D imaging, planning, and image-guided navigation are critical adjuncts when performing iliosacral osteosynthesis in the presence of existing sacropelvic hardware. Although SI joint hypermobility could be a risk factor for sacral nonunion in this population, more studies are needed to better understand the role of SI joint stabilization and fusion in the treatment of sacral fractures.
